# Role of Interleukin 17 in Arthritis Chronicity through Survival of Synoviocytes via Regulation of Synoviolin Expression

**DOI:** 10.1371/journal.pone.0013416

**Published:** 2010-10-15

**Authors:** Myew-Ling Toh, Gaelle Gonzales, Marije I. Koenders, Anne Tournadre, David Boyle, Erik Lubberts, Yuan Zhou, Gary S. Firestein, Wim B. van den Berg, Pierre Miossec

**Affiliations:** 1 Research Unit Immunogenomics and Inflammation, EA 4130, Hospital Edouard Herriot, University of Lyon, Lyon, France; 2 Rheumatology Research and Advanced Therapeutics, Radboud University Nijmegen Medical Center, Nijmegen, The Netherlands; 3 Division of Rheumatology, Allergy and Immunology, Department of Medicine, University of California San Diego, La Jolla, California, United States of America; New York University, United States of America

## Abstract

**Background:**

*T*he use of TNF inhibitors has been a major progress in the treatment of chronic inflammation. However, not all patients respond. In addition, response will be often lost when treatment is stopped. These clinical aspects indicate that other cytokines might be involved and we focus here on the role of IL-17. In addition, the chronic nature of joint inflammation may contribute to reduced response and enhanced chronicity. Therefore we studied the capacity of IL-17 to regulate synoviolin, an E3 ubiquitin ligase implicated in synovial hyperplasia in human rheumatoid arthritis (RA) FLS and in chronic reactivated streptococcal cell wall (SCW)-induced arthritis.

**Methodology/Principal Findings:**

Chronic reactivated SCW-induced arthritis was examined in IL-17R deficient and wild-type mice. Synoviolin expression was analysed by real-time RT-PCR, Western Blot or immunostaining in RA FLS and tissue, and p53 assessed by Western Blot. Apoptosis was detected by annexin V/propidium iodide staining, SS DNA apoptosis ELISA kit or TUNEL staining and proliferation by PCNA staining. IL-17 receptor A (IL-17RA), IL-17 receptor C (IL-17-RC) or synoviolin inhibition were achieved by small interfering RNA (siRNA) or neutralizing antibodies. IL-17 induced sustained synoviolin expression in RA FLS. Sodium nitroprusside (SNP)-induced RA FLS apoptosis was associated with reduced synoviolin expression and was rescued by IL-17 treatment with a corresponding increase in synoviolin expression. IL-17RC or IL-17RA RNA interference increased SNP-induced apoptosis, and decreased IL-17-induced synoviolin. IL-17 rescued RA FLS from apoptosis induced by synoviolin knockdown. IL-17 and TNF had additive effects on synoviolin expression and protection against apoptosis induced by synoviolin knowndown. In IL-17R deficient mice, a decrease in arthritis severity was characterized by increased synovial apoptosis, reduced proliferation and a marked reduction in synoviolin expression. A distinct absence of synoviolin expressing germinal centres in IL-17R deficient mice contrasted with synoviolin positive B cells and Th17 cells in synovial germinal centre-like structures.

**Conclusion/Significance:**

IL-17 induction of synoviolin may contribute at least in part to RA chronicity by prolonging the survival of RA FLS and immune cells in germinal centre reactions. These results extend the role of IL-17 to synovial hyperplasia.

## Introduction

Anti-TNF therapies have an established role in rheumatoid arthritis (RA) treatment however in the clinic RA remains a chronic disease with up to 40% nonresponders and subsequent flares when TNF inhibitors are ceased. RA is characterised by chronic synovial inflammation and hyperplasia. Synovial hyperplasia is a consequence of an imbalance between proliferation and apoptosis of resident fibroblast-like synoviocytes (FLS). Resistance of cells to apoptosis is partly due to the upregulation of molecules counteracting the apoptosis cascade. There is substantial evidence that synovial hypertrophy contributes to the chronicity of RA with ultimately destructive consequences. Joint synovectomy by surgical, chemical or radiation measures, removes the pathologic synovial tissue burden leading to cessation of arthritis and more prolonged remission [1]. Similarly to cancer cells, RA FLS demonstrate dysregulated proliferation and apoptosis, anchorage independent growth, intrinsically express proto-oncogenes and functional mutations in tumour suppressor genes such as p53 and are capable of spontaneously invading human cartilage [2].

Up to 40% of RA patients are nonresponders to current anti-TNF biologics suggesting that other molecules implicated in synovial inflammation and/or hyperplasia may contribute to disease chronicity [3]. Recently, synoviolin a novel E3 ubiquitin ligase present in the endoplasmic reticulum, was identified in RA FLS and synovial lining tissue, and may be implicated in dysregulated proliferation and apoptosis in RA [4]. The overexpression of synoviolin leads to destructive arthritis in mice [4]. In murine arthritis, synoviolin heterozygous knockout mice are protected against arthritis development associated with reduced synovial hypercellularity. In addition, synoviolin is increased during the course of murine collagen induced arthritis (CIA) [5]. In contrast to murine studies, the role of synoviolin in human RA is less well understood. In vitro, synoviolin has been reported to protect against both ER-stress-induced apoptosis in RA FLS [4] and p53-mediated apoptosis in 293 cells [6]. In RA, the proinflammatory cytokines IL-1 and TNF derived predominantly from synovial macrophages lead to sustained synoviolin expression [3], [5], [7]. In vivo, synoviolin has been reported to be elevated in a subset of circulating immune cell subsets which may be associated with anti-TNF nonresponse [3].

IL-17 is a pleiotropic proinflammatory cytokine produced from Th17 cells [8]. IL-17A is the prototypic member belonging to a family of 6 ranging from IL-17A to IL-17F [9]. IL-17A mediates its biological effects through binding to a receptor complex consisting of IL-17RA and IL-17RC subunits which are expressed in RA FLS [10]. The IL-17 receptor complex interacts with a STIR (SEFIR, similar expression to fibroblast growth factor genes) subdomain leading to activation of act1, TRAF6, nuclear factor kappa B (NFκB), activator protein 1 (AP-1) and mitogen protein kinases (MAPK) [10], [11], [12], [13], [14]. IL-17 has a critical role in RA joint inflammation and destruction [15]. IL-17 has been described to be implicated in synovial hypercellularity in murine arthritis models [16]. In addition, IL-17RC has been reported to regulate apoptosis [17]. The role of T-cell derived cytokines such as IL-17 in regulation of dysregulated apoptosis and proliferation in RA FLS is not known. In this study, we demonstrate that IL-17R-mediated induction of synoviolin contributes to chronic synovial hyperplasia and inflammation by prolonging the survival of RA FLS and germinal centre reactions.

## Results

### Synoviolin induction by IL-17 in RA FLS

**Figure 1:**
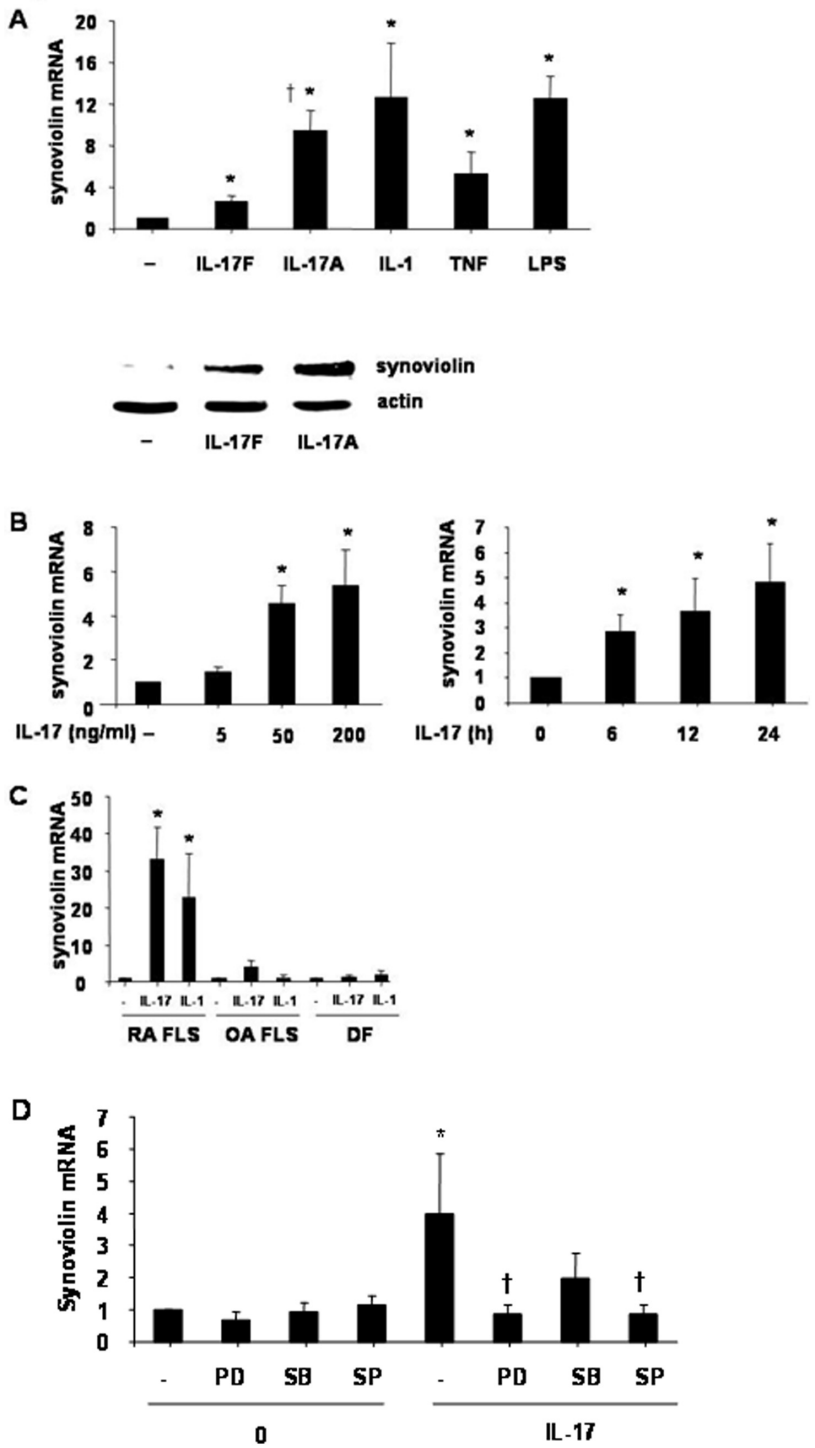
Effect of IL-17 on synoviolin expression in RA FLS. RA FLS were stimulated with IL-17A or IL-17F (50 ng/ml), IL-1 10 ng/ml, TNF 10 ng/ml or LPS 100 ng/ml for 24 h and synoviolin mRNA expression measured by real-time RT-PCR (**A, upper panel**). The results based on a ratio of synoviolin/β-actin mRNA amplification are presented as the fold induction in synoviolin mRNA expression relative to control samples, n = 3, mean ± SEM. * *P*<0.05 compared to no treatment control. RA FLS were treated with IL-17A or IL-17F (50 ng/ml) for 24 h and synoviolin expression measured by Western Blotting (**A, lower panel**). The membrane was stripped and reprobed for β-actin and the result is representative of n = 3. RA FLS were treated with IL-17 5-200 ng/ml for 24 h (**B, left panel**) or IL-17 50 ng/ml over 24 h (**B, right panel**) and synoviolin expression measured by real-time RT-PCR, n = 3, mean ± SEM, * *P*<0.05. Synoviolin mRNA expression in RA FLS, OA FLS and OA dermal fibroblasts that were untreated (–) or were treated with 50 ng/ml or 10 ng/ml of IL-1 for 24 h **(C).** * *P*<0.05 versus **untreated FLS.** RA FLS were preincubated with PD98059 (50μM), SB203580 (5μM) or SP600125 (50μM) for 1 hour, then treated with IL-17 50 ng/ml for 1 hour. Synoviolin expression was measured by real-time PCR, mean ± SEM, n = 3 (**D**). * p<0.05 compared to IL-17 treatment.

We investigated the expression of synoviolin in cultured RA FLS treated with IL-17A or IL-17F compared to IL-1, TNF and LPS by real time RT-PCR and Western blot. Synoviolin was constitutively expressed in RA FLS, and induced by IL-17A, IL-17F, IL-1 or TNF at 24 h (Figure 1A, upper panel). The induction of synoviolin expression by IL-17A at 12 h was weaker than induction by IL-1β or LPS but stronger than IL-17F or TNF-α. IL-17-induced synoviolin expression was confirmed at the protein level. IL-17A was a more potent inducer of synoviolin compared to IL-17F (Figure 1A, lower panel).

Given that IL-17A induced more robust synoviolin induction than IL-17F, subsequent experiments were carried out with IL-17A alone and hereby referred to as IL-17. We next determined the dose range of synoviolin induction by IL-17. A dose dependent increase in synoviolin mRNA by IL-17A was observed (Figure 1B, left panel). The timecourse of IL-17 50 ng/ml induction of synoviolin was examined over 24 hours. IL-17A induced significant synoviolin mRNA expression at 6 h which increased and remained elevated over 24 h (Figure 1B, right panel). IL-17A increased synoviolin mRNA 4.9 fold at 24 h (*P*<0.05). In comparison with OA FLS or OA dermal fibroblasts, IL-17-induced synoviolin expression was more marked in RA FLS (Figure 1C).

### ERK and JNK activation is required for IL-17 induced expression of synoviolin

ERK MAPK and ETS1 transcription factor activation have been described to modulate synoviolin expression in murine cells[5]. To investigate the involvement of MAPK activation in IL-17 induced synoviolin expression, RA FLS were preincubated with MAPK inhibitors for 1 hour [18], [19] and then treated with IL-17 50 ng/ml over 12 h. Inhibition of ERK (PD98059) or JNK (SP600125) was associated with a marked reduction in synoviolin mNA expression (Figure 1D). Although there was a trend, we did not observe a statistically significant reduction in IL-17 induced synoviolin expression in the presence of the p38 pathway inhibitor (SB203580).

### IL-17 protects RA FLS from SNP-induced apoptosis

**Figure 2:**
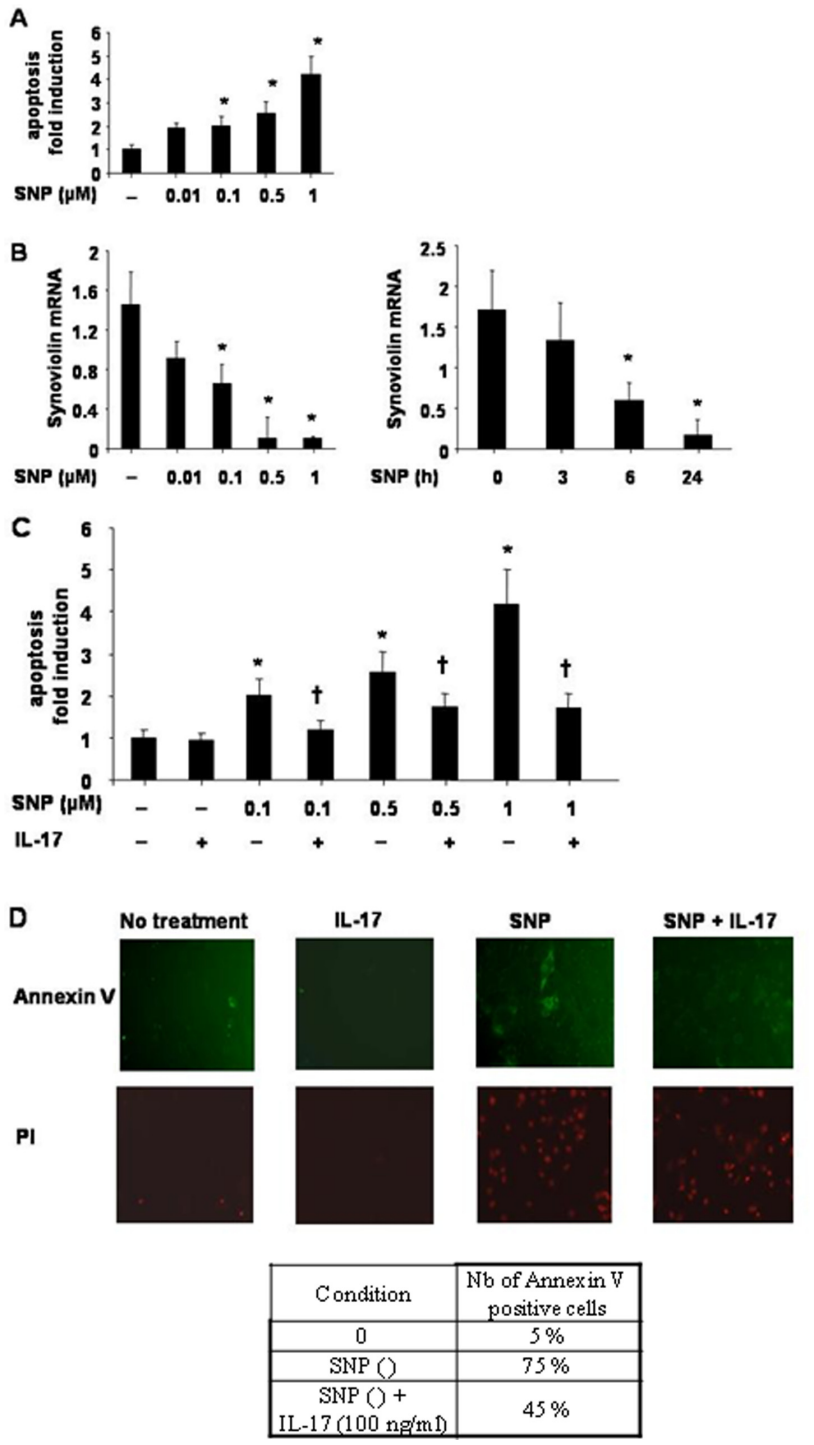
Effect of IL-17 on RA FLS apoptosis. RA FLS were treated with SNP 0.01-1 µM for 24 h and apoptosis measured by SS DNA apoptosis kit (**A**). The results are presented as the fold induction in apoptosis relative to control samples, n = 3, mean ± SEM of duplicate experiments from 3 different RA donors. * *P*<0.05 compared to no treatment control. RA FLS were treated with SNP 0.01–1 µM for 24 h (**B, left panel**) or 0.1 µM over 24 h (**B, right panel**) and synoviolin expression measured by real-time RT-PCR. The results are expressed as the ratio of synoviolin/β-actin mRNA amplification, n = 3, mean ± SEM of duplicate experiments from 3 different RA donors. * *P*<0.05 compared to no treatment control. RA FLS were pretreated with IL-17 100 ng/ml for 2 h then cotreated with SNP 0.1-1 µM for 24 h and apoptosis measured by SS DNA apoptosis kit (**C**). The results are presented as the fold induction in apoptosis relative to control samples, n = 3, mean ± SEM of duplicate experiments from 3 different RA donors. * *P*<0.05 compared to no treatment control. RA FLS were treated as above and annexin V analysed. Representative serial fluorescent microscopic pictures of Annexin V positive cells (green), double labelled with Propidium iodide (PI, red) at magnification x 200 (**D**).

To determine the functional significance of IL-17-induced synoviolin expression in RA FLS, we examined the effects of IL-17 treatment on SNP-induced apoptosis. SNP induces apoptosis by NO donation and leads to cell death by both ER-stress and p53-mediated apoptosis [20], [21], [22]. RA FLS demonstrated low rates of spontaneous apoptosis. SNP (0.1–1 μM) induced a significant increase in apoptosis up to 70–80% of annexin V positive cells by FACs analysis (data not shown) or by SS DNA fragmentation, associated with decreased synoviolin expression at 6 and 24 h (Figure 2A, B, *P*<0.05). Treatment of RA FLS by IL-17 over 24 h significantly impaired SNP-induced apoptosis (Figure 2C). This effect of IL-17 was confirmed by a reduction in SNP-induced annexin V positive cells (Figure 2D, *P*<0.05).

### Effect of IL-17RA or IL-17RC RNA interference on RA FLS apoptosis

**Figure 3:**
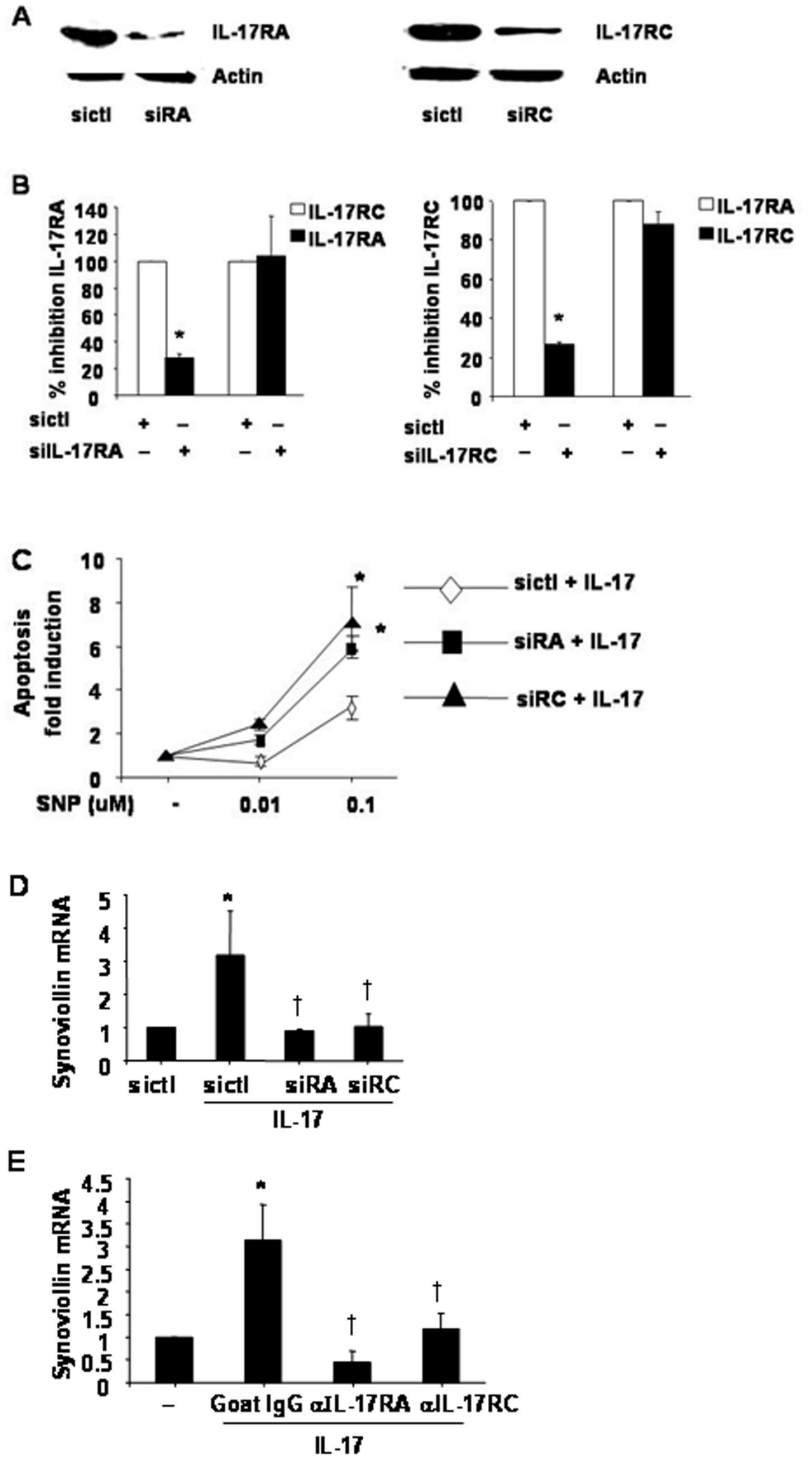
Effect of IL-17RA or IL-17RC knockdown on apoptosis, synoviolin expression in RA FLS. **To confirm specificity of siRNA knockdown of individual IL-17 receptors,** RA FLS were nucleofected (amaxa) for 24 h with 0.5 µg IL-17RA (siRA), 0.05 µg IL-17RC (siRC) or siCONTROL siRNA (sictl) serum starved overnight then treated 50 ng/ml IL-17A for 8 h. IL-17RA or IL-17RC protein expression was measured by Western Blot. Membranes were stripped and reprobed for actin as a loading control (**A**). IL-17RA or IL-17RC mRNA expression was measured by real-time RT-PCR, normalised to GAPDH and expressed as percentage inhibition compared to sictl siRNA (**B**). * *P*<0.05, compared to sictl nucleofected RA FLS. RA FLS were nucleofected as above, serum starved overnight then pretreated with 50 ng/ml IL-17A for 2 h then cotreated with SNP 0.1 µM overnight and apoptosis measured by SS DNA apoptosis assays expressed as fold induction of apoptosis (**C**), n = 3 from 3 separate RA donors, mean ± SEM of duplicate experiments. * *P*<0.05, compared to sictl nucleofected RA FLS. RA FLS were nucleofected as above serum starved overnight then treated with 50 ng/ml IL-17A and (**D**) synoviolin levels analysed by real-time RT-PCR. Synoviolin mRNA level was normalized to GAPDH and expressed as fold increase over sictl siRNA treated samples, n = 5, mean ± SEM of duplicate experiments. * *P*<0.05, compared to sictl nucleofected RA FLS. † *P*<0.05 compared to IL-17 sictl treated samples. RA FLS were pretreated with 2 µg/ml of anti-IL-17RA or anti-IL-17RC Abs or isotype control goat Abs for 2 h then cotreated with IL-17 50 ng/ml for 24 h and synoviolin expression measured by real-time RT-PCR (**E**) , n = 3, mean ± SEM. * *P*<0.05, compared to untreated control. † *P*<0.05 compared to IL-17 treated samples.

To confirm a protective role for IL-17R signaling in SNP-induced apoptosis we next examined the effect of IL-17RA or IL-17RC RNA interference in RA FLS. IL-17RA siRNA lead to a 72% inhibition of IL-17RA mRNA and protein expression, whereas IL-17RC siRNA inhibited IL-17RC mRNA and protein expression by 70% (Figure 3A, B). To determine the specificity of RNA interference we examined the effects of IL-17RA or IL-17RC interference on IL-17RA or IL-17RC expression. IL-17RA RNA interference had either no effect or slightly increased IL-17RC mRNA expression (Figure 3B). IL-17RC RNA interference had no effect on IL-17RA mRNA expression (Figure 3B). In the presence of IL-17, IL-17RA or IL-17RC RNA interference significantly enhanced SNP 0.1 μM induced apoptosis in RA FLS, with a 3 or 2.7 fold increase in apoptosis respectively compared to sicontrol (Figure 3C, *P*<0.05), confirming a protective effect of IL-17 on RA FLS apoptosis.

### Effect of IL-17RA or IL-17RC RNA interference on synoviolin expression

We next determined the effects of IL-17RA or IL-17RC RNA interference on synoviolin expression. Both IL-17RA or IL-17RC siRNA lead to near complete abrogation of IL-17-induced synoviolin expression in RA FLS (Figure 3D). As expected IL-17RA or IL-17RC RNA interference antagonism also reduced IL-17-mediated p65 induction, whereas IL-17RA RNA interference more potently suppressed IL-17-induced c-fos expression compared to IL-17RC (data not shown). We confirmed IL-17R-mediated modulation of synoviolin by the use of neutralising Abs against IL-17RA or IL-17RC [23]. Anti-IL-17RA or anti-IL-17RC Abs significantly antagonised IL-17-induced synoviolin expression (Figure 3E, *P*<0.05).

### Effect of synoviolin RNA interference on RA FLS apoptosis

**Figure 4:**
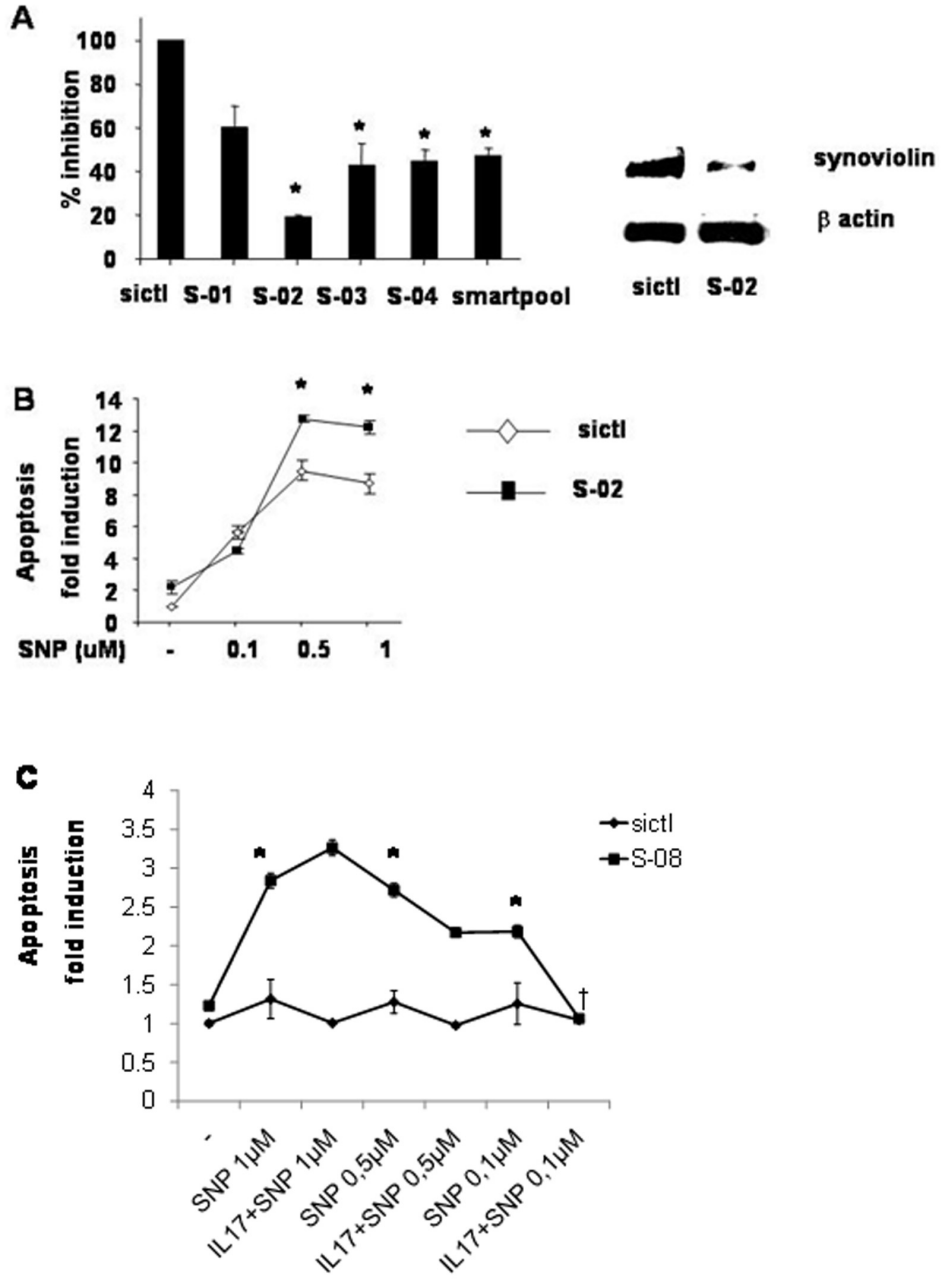
Effect of synoviolin knockdown on apoptosis in RA FLS. RA FLS were nucleofected (amaxa) for 24 h with 0.5 µg of 4 synoviolin siRNA duplexes (S-01 to S-04), synoviolin smartpool siRNA duplex or siCONTROL siRNA (sictl) and synoviolin expression analysed by real-time RT-PCR **(A, left panel)** or Western Blot **(A, right panel).** Synoviolin mRNA was normalized to GAPDH and results expressed as the percentage fold reduction compared to sictl treated samples, n = 5, mean ± SEM of duplicate experiments. * *P*<0.05, compared to sictl nucleofected RA FLS. (**B**), RA FLS were nucleofected as above then treated with SNP 0.1–1 µM overnight and apoptosis measured by the SS DNA apoptosis assay. Results are expressed as fold induction in apoptosis, n = 3 from 3 separate RA donors, mean ± SEM of duplicate experiments. * *P*<0.05, compared to sictl nucleofected RA FLS. (**C**), RA FLS were nucleofected as above, serum starved overnight then pretreated with 100 ng/ml IL-17A for 2 h then cotreated with SNP 0.1–1 µM overnight and apoptosis measured by SS DNA apoptosis assays expressed as fold induction of apoptosis, n = 3 from 3 separate RA donors, mean ± SEM of duplicate experiments. * *P*<0.05, compared to sictl nucleofected RA FLS. † *P*<0.05 compared to SNP treatment in S-08 nucleofected RA FLS.

To determine if synoviolin may inhibit SNP-induced apoptosis in RA FLS, we determined effects of synoviolin RNA interference in RA FLS. We confirmed 80% knockdown of synoviolin mRNA and protein by RNA interference in RA FLS (Figure 4A). Duplex 2 (S-02) was the most efficient and was chosen for subsequent experiments. Synoviolin RNA interference increased SNP-induced apoptosis by 4 fold in RA FLS (Figure 4B, *P*<0.05) demonstrating the specificity of its contribution. To confirm that the anti-apoptotic effects of IL-17 were mediated by synoviolin, we demonstrated that the protective effects of IL-17 were completely abrogated in the presence of synoviolin knockdown at the lowest dose of SNP (Figure 4C, *P*<0.05). However, at higher concentrations of SNP the anti-apoptotic effects of IL-17 could be reversed.

### Effect of IL-17 and TNF on synoviolin expression and apoptosis in RA FLS

**Figure 5:**
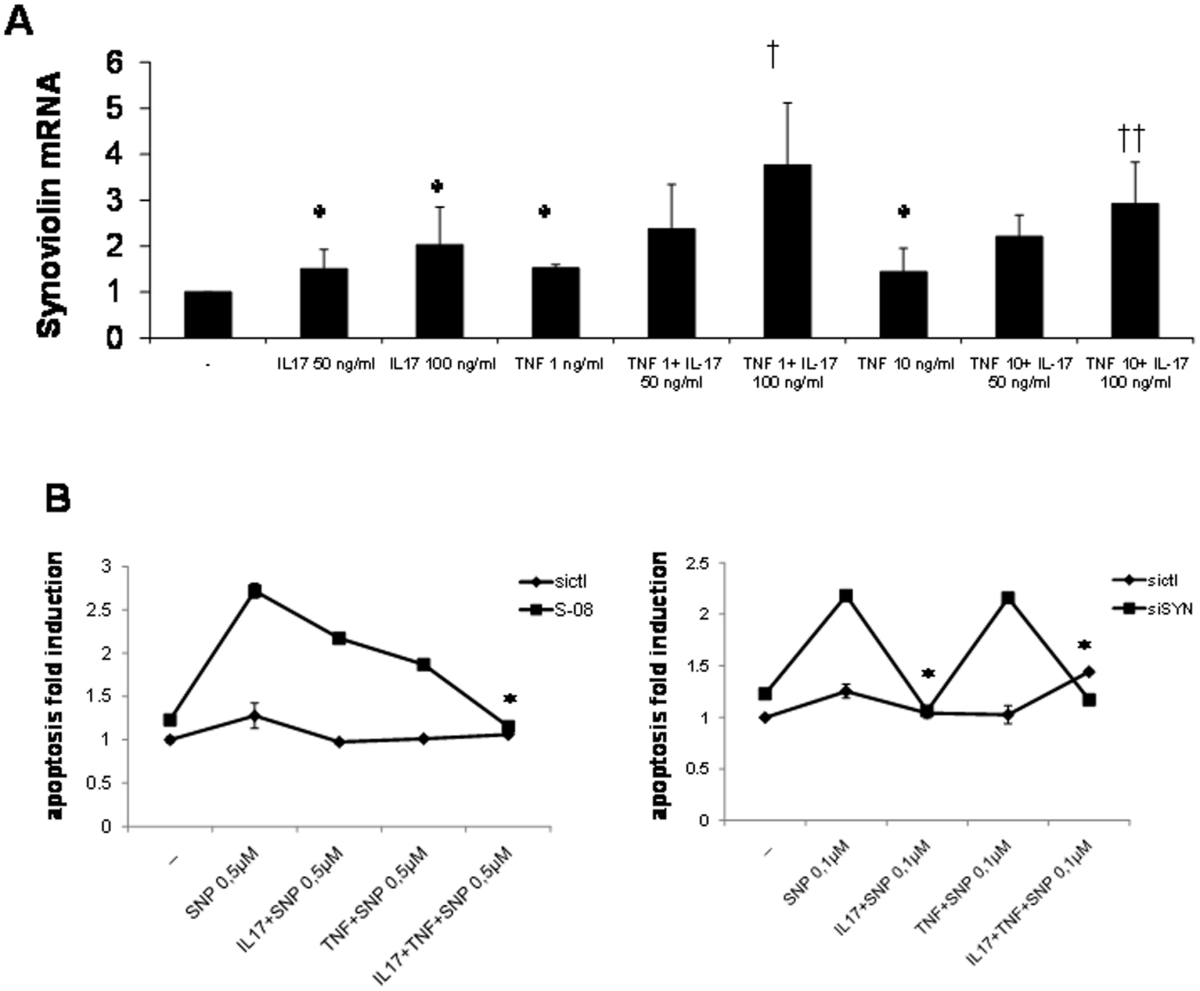
Effect of IL-17 and TNF on RA FLS synoviolin expression and apoptosis. RA FLS were stimulated with IL-17A (50 or 100 ng/ml) and/or TNF (1 or 10 ng/ml) for 24 h and synoviolin mRNA expression measured by real-time RT-PCR (**A**). The results based on a ratio of synoviolin/β-actin mRNA amplification are presented as the fold induction in synoviolin mRNA expression relative to control samples, n = 3, mean ± SEM. * *P*<0.05 compared to no treatment control. † *P*<0.05 compared to TNF treated samples. RA FLS were pretreated with IL-17 100 ng/ml and TNF 10 ng/ml for 2 h then cotreated with SNP 0.5 µM (**B, left panel**) or 0.1 µM (**B, right panel**) for 24 h and apoptosis measured by SS DNA apoptosis kit. The results are presented as the fold induction in apoptosis relative to control samples, n = 3, mean ± SEM of duplicate experiments from 3 different RA donors. * *P*<0.05 compared to SNP treatment in S-08 nucleofected RA FLS.

IL-17 and TNF have synergistic or additive effects on many of the biological activities of IL-17 [24]. As noted previously TNF had a weaker effect on induction of synoviolin than IL-17A. IL-17 100 ng/ml in combination with TNF (1 or 10 ng/ml) had additive effects on synoviolin induction in comparison with TNF alone (*P*<0.05), but only trended to significance compared to IL-17 alone (Figure 5A).

Similarly, at the higher concentrations of SNP 0.5 µM (or 1 µM data not shown) only the combination of IL-17 and TNF was capable of completely abrogating RA FLS apoptosis in the presence of synoviolin knockdown compared to either cytokine alone (Figure 5B). At the lowest concentration of SNP 0.1 µM, IL-17 but not TNF was capable of completely protecting RA FLS from apoptosis induced by synoviolin knockdown. Some variability in the potency of cytokine-induced synoviolin induction and apoptosis was noted between different cell lines.

### Reduced synoviolin expression in IL-17R deficient mice during chronic SCW-induced arthritis

**Figure 6:**
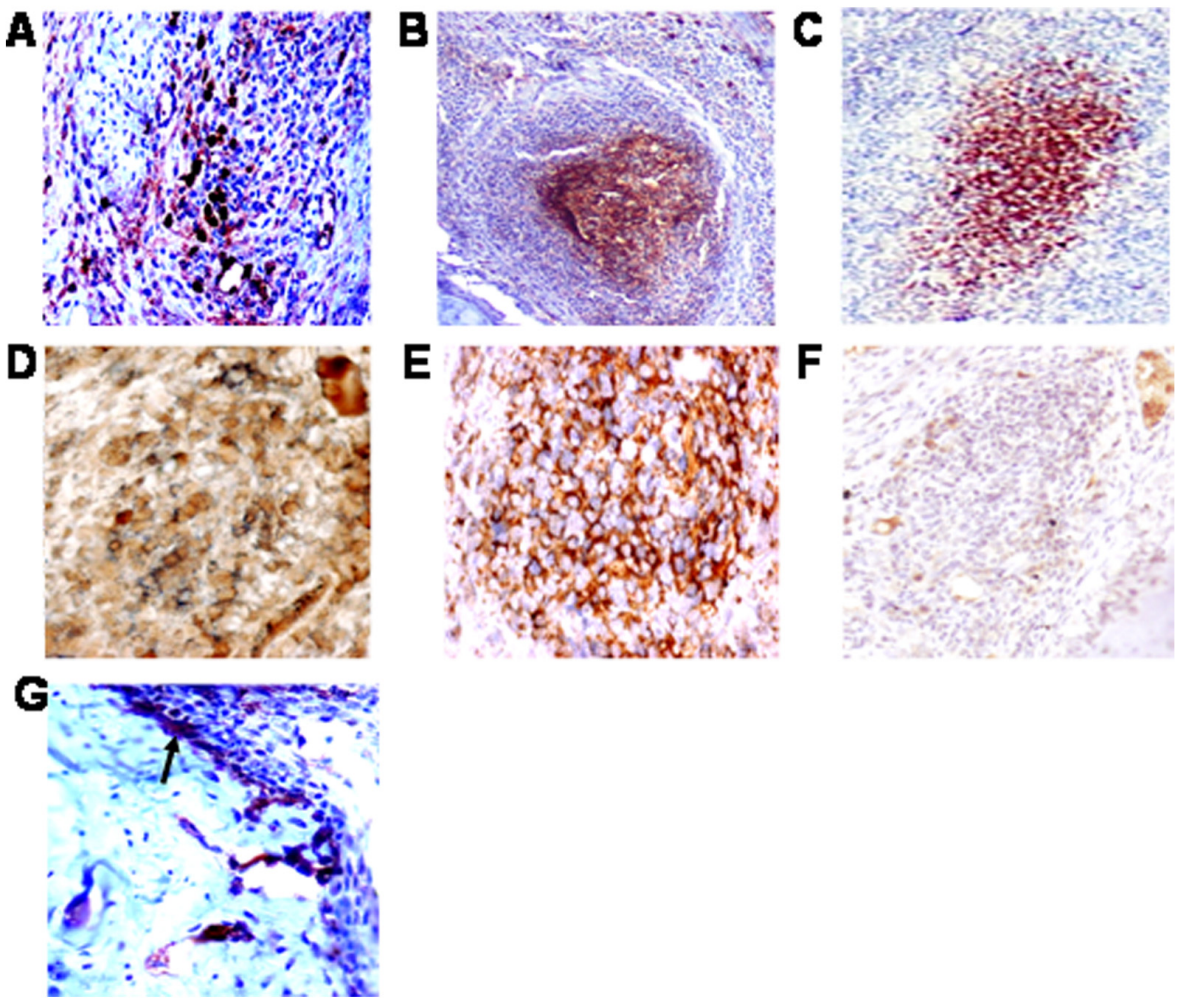
Synoviolin expression during chronic streptococcal cell wall-induced arthritis in wild-type (WT) mice. A representative image of synoviolin-positive staining diffusely (**A**) or in follicle-like structures of synovium (**B** and **C**, magnification X 200). Double immunostaining showing colocalisation of synoviolin (brown) and CD20 (blue) staining (**D**, magnification X 600) or IL-17 (blue) staining (**E**, magnification X 600) or isotype control (**F**, magnification X 600) in follicle-like structures of arthritic knee joints from WT mice. Synoviolin staining in synoviocytes infiltrating cartilage at the pannus-cartilage junction (**G**, magnification X 400).

To confirm the requirement of IL-17R-dependent signaling in IL-17-induced synoviolin expression we examined the effects of IL-17R deficiency on synoviolin expression. We first assessed synoviolin expression histologically in injected knee joints of WT mice during chronic SCW-induced arthritis. The synoviolin positive cells in murine synovium were observed either diffusely or in some sections almost entirely limited to follicle-like structures. Serial sections of follicle-like structures, demonstrated that synoviolin positive cells colocalised with IL-17 and CD19 positive staining suggesting that synoviolin was expressed by both Th17 cells and CD19+ B cells (Figure 6A-E, isotype control 6F). Synoviolin expression was also observed in RA FLS invading cartilage at the pannus-cartilage junction in WT mice (Figure 6G).

**Figure 7:**
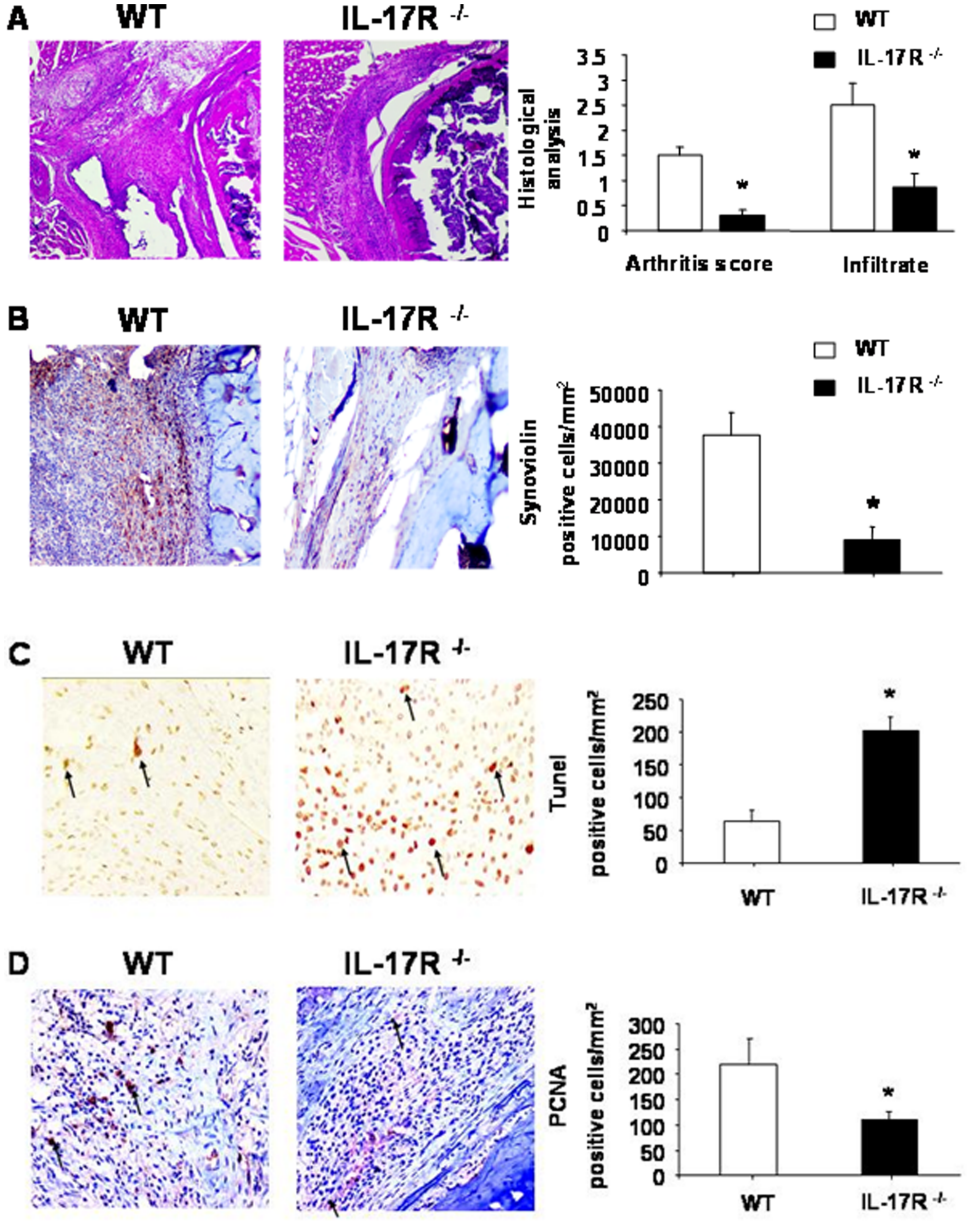
Severity of chronic streptococcal cell wall-induced arthritis in wild-type (WT) or IL-17R ^−/−^ mice A). (A representative image of haematoxylin and eosin-stained synovial knee joint sections in WT or IL-17R **^−^** ^/**−**^ mice at day 42 after 5 repeated injections of streptococcal cell wall (SCW) fragments (magnification ×40). Arthritis severity and inflammatory infiltrate were scored histologically in injected joints of WT or IL-17R **^−^** ^/**−**^ mice. Arthritis severity was scored on a scale of 0–2 and synovial infiltrate on a scale of 0–3. Results are expressed as mean ± SEM of 2 separate experiments, n = 10 mice per group per experiment, * *P*<0.05 compared to WT mice. **Synoviolin expression is reduced in IL-17R ^−/−^ mice with chronic SCW-induced arthritis** (**B**). Synoviolin expression in sections of arthritic knee joints from, a wild-type (WT) or IL-17R **^−^** ^/**−**^ mouse after 5 repeated injections of SCW fragments (magnification x 40, day 42, n = 10 mice in WT or IL-17R **^−^** ^/**−**^ groups from 2 separate experiments). Synoviolin expression was quantified in 10 high power fields, averaged and expressed as the number of synoviolin positive cells/mm^2^, * *P*<0.05 compared to WT mice. **Synovial apoptosis in WT or IL-17R ^−/−^ mice with chronic streptococcal cell wall-induced arthritis** (**C**). A representative image of increased TUNEL staining in IL-17R **^−^** ^/**−**^ synovium (magnification ×200.). The number of TUNEL-positive cells was quantified in 10 high power fields, averaged and expressed as the number of TUNEL positive cells/mm^2^, mean ± SEM, * *P*<0.05 compared to WT mice. **Synovial PCNA staining in knee joint sections from WT or IL-17R ^−/−^ mice with chronic streptococcal cell wall-induced arthritis** (**D**). A representative image of PCNA staining in synovial sections (magnification ×200). The number of PCNA-positive cells was quantified in 10 high power fields, averaged and expressed as the number of PCNA positive cells/mm^2^, mean ± SEM, * *P*<0.05 compared to WT mice.

As previously described [25], compared to WT mice, IL-17R ^−/−^ mice had reduced synovial hypercellularity leading to significantly decreased macroscopic arthritis severity scores (Figure **7**A, mean ± SEM score 1.5±0.17 in WT versus 0.31±0.10 in IL-17R ^−/−^ mice; *P*<0.05). Similarly, in comparison to WT animals, IL-17R ^−/−^ mice histologically displayed markedly reduced inflammatory infiltrate scores (mean ± SEM score 2.5±0.43 in WT versus 0.87±0.27 in IL-17R ^−/−^ mice ; *P*<0.05). Synoviolin expression was reduced in the synovial knee sections of IL-17R ^−/−^ compared to WT mice (Figure **7**B). In IL-17R ^−/−^ mice, synoviolin expression was either weak or limited throughout the inflammatory infiltrate.

We next examined synovial apoptosis by TUNEL staining of synovial knee joints from mice with chronic SCW-induced arthritis. TUNEL staining was significantly increased in synovial knee joints from IL-17R ^−/−^ mice than from WT mice (mean ± SEM TUNEL-positive cells per high-power field 203±21 versus 63±18; *P*<0.05, Figure **7**C).

In situ proliferation was detected by PCNA staining. In WT mice with chronic SCW-induced arthritis, the number of proliferating cells detected was 219±52 PCNA-positive cells ± SEM per high-power field which was further reduced in IL-17R ^−/−^ mice compared to WT mice (110±15, Figure **7**D).

## Discussion

The chronicity of RA disease is not well understood. Persistence of biological abnormalities in RA FLS or synovial tissue despite disease-modifying treatment is supported by a substantial proportion of nonresponders and the transient effects of current gold standard treatment such as anti-TNF biologics. New treatment regimens will need to go beyond the simple control of inflammation if more prolonged remissions are to be achieved. Synoviolin is an E3 ubiquitin ligase implicated in murine arthritis pathogenesis. Overexpression of synoviolin leads to spontaneous destructive arthritis, and synoviolin ^−/+^ mice are resistant to CIA [4]. The protective role of synoviolin in ER stress-induced apoptosis and cell survival has been demonstrated in RA FLS and other cell types [26], [27], [28], [29]. In RA FLS, we and others have demonstrated that synoviolin plays an important role in RA FLS growth [4]. Synoviolin knockdown augments ER-stress induced apoptosis and diminishes intrinsic and cytokine-induced FLS proliferation. We recently observed expression of synoviolin in T cells, macrophages and RA FLS, which is increased by the predominantly macrophage-derived cytokines IL-1 and TNF known to regulate RA FLS cell growth and survival [3]. In contrast, IL-17 derived from Th17 cells has not been reported to modulate RA FLS cell growth. Indeed, reports in the literature suggest that IL-17 regulates cell growth in only certain cell types such as lymphocytes and data in cancer is controversial. In this study, IL-17 exerts an anti-apoptotic effect, mediated by IL-17RA and IL-17RC, associated with increased synoviolin expression. These data suggest that IL-17 contributes to RA chronicity through both synovial inflammation and hyperplasia.

An anti-apoptotic role for IL-17 is supported by data in IL-17R knockout mice associated with markedly reduced synovial hypercellularity [30]. Other proflammatory cytokines such as TNF [31] and MIF [32] have been reported to contribute to synovial hyperplasia and the increased survival of RA FLS. IL-17 rescued RA FLS from SNP-induced apoptosis and IL-17RA or RC siRNA augmented SNP-induced apoptosis. An important role for IL-17RC in protection against apoptosis has been reported in a prostate cancer line whereby IL-17RC inhibits caspase 2, 8 and subsequently caspase 3 [17]. In contrast an anti-apoptotic effect of IL-17RA has not previously been described. Similarly both IL-17R components were involved in IL-17 induced p65 NFκB and c-fos AP-1 activation in RA FLS. This is consistent with human and murine data demonstrating the requirement for both IL-17RA and IL-17RC subcomponents in the biological effects of IL-17 [23], [33]. In contrast to the clear anti-apoptotic effects of IL-17, IL-17 (1-100 ng/ml) over 6 h, 24 h, 48 h or 7 days did not significantly stimulate RA FLS proliferation (data not shown).

We observed that IL-17R mediated ERK and JNK activation were required in RA FLS synoviolin expression. Cytokine induced ERK MAPK and Ets 1 transcription factor have been reported to control synoviolin expression in mouse cells [5]. In contrast to IL-17-dependent synoviolin in human RA FLS, in mouse cells Gao et al [5] demonstrated that JNK activation is dispensable for IL-1β-dependent synoviolin expression. The differences observed may be cytokine dependent and/or associated with mouse/human cell differences. NFκB binding sites are not present in the synoviolin promoter. These data suggest that IL-17R-induced synoviolin expression via ERK and JNK MAPK activation contributes to protection against apoptosis in RA FLS. MAPK and AP-1 activation have an established role in cell survival.

The underlying mechanism for IL-17-mediated protection against RA FLS apoptosis may be associated at least in part to IL-17 induced synoviolin expression. Synoviolin RNA interference enhances ER stress induced apoptosis in RA FLS[34], and in this study synoviolin siRNA augments SNP-induced RA FLS apoptosis. Herein, IL-17 treatment subsequently reduced synoviolin siRNA-induced RA FLS apoptosis, rescued RA FLS from SNP-induced apoptosis and dose dependently induced synoviolin expression. Furthermore, either IL-17 or synoviolin inhibition enhanced SNP-induced apoptosis. Complete abrogation of synoviolin knockdown-induced apoptosis at high doses of SNP required the combination of both IL-17 and TNF consistent with the additive effects of this cytokine combination on synoviolin induction. IL-17 had more potent effects than TNF on synoviolin induction and protection against RA FLS apoptosis. Thus, in addition to additive effects of IL-17 and TNF on inflammation and bone destruction this cytokine combination enhanced anti-apoptotic effects of IL-17. In the mixed cytokine milieu of an inflammed arthritic joint, other proinflammatory cytokines may enhance the protective effects of IL-17 against FLS apoptosis implicated in synovial hyperplasia contributing to pannus persistence and chronicity. It is possible that IL-17 may also exert its anti-apoptotic effects in a synoviolin independent manner such as through induction of NFκB, MAPK and AP-1. Both endogenous and cytokine induced synoviolin expression in RA FLS promote RA FLS survival.

It is unclear how IL-17-induced or endogenous synoviolin expression may be anti-apoptotic in RA FLS. Recent data suggests that the tumour suppressor gene, p53 may be regulated by synoviolin [6]. Synoviolin binds to, ubiquitinates and degrades cytoplasmic p53. Furthermore, IL-17 rescued RA FLS from SNP-induced apoptosis. SNP is a nitric oxide (NO) donor, and elevated NO levels play a key role in apoptosis of cells involved in the inflammatory response. NO mediates apoptosis by both p53-mediated apoptosis [35] and ER-stress induced apoptosis [36], [37]. Although it is tempting to speculate that IL-17 may indirectly modulate p53 expression through synoviolin expression we were unable to confirm this hypothesis at least at timepoints up to 24 h of IL-17 treatment (data not shown). IL-17 did not suppress p53 protein expression in timepoints up to 24 h despite rescue of RA FLS from SNP-induced apoptosis by IL-17, associated with increased synoviolin expression. IL-17 increased p53 expression prior to synoviolin expression at 4 h and 6 h respectively following IL-17A treatment which was sustained out to 24 h (data not shown). However, the time course needs to be extended to examine whether p53 degradation occurs following synoviolin induction beyond 24 h. Moreover, synoviolin sequesters p53 in the ER. It would be interesting to examine p53 sequestration following IL-17-induced synoviolin upregulation, and the ubiquitination status of p53 at later timepoints. Although, IL-17 was capable of inducing both synoviolin and p53 the net effect was anti-apoptotic. Other proinflammatory cytokines such as MIF have clear protective effects against SNP-induced apoptosis in RA FLS through marked downregulation of p53 expression [32].

Interestingly we identified synoviolin and IL-17 coexpressing cells in synovial germinal centre and follicle-like structures in WT mice with SCW-induced arthritis. Th17 cells have been recently described to play a key role in germinal centre formation [38]. We previously described synoviolin expression in peripheral blood CD3 + T cells from RA patients [3]. In addition, such synoviolin expressing Th17 cells were found in close contact with synoviolin expressing CD19 + B cells in follicle-like structures of these mice. Subsequent to this finding we cell sorted human CD19 + B cells from the peripheral blood of RA patients and found that synoviolin was also highly expressed in the blood (data not shown). Germinal centre-like structures including synoviolin expression were completely absent in IL-17R deficient mice. IL-17R signaling has been demonstrated to be critical for the formation of such structures in an autoimmune model of arthritis as in autoimmune BXD2 mice [38]. In this study the coexistence of Th17 cells and B cells expressing synoviolin suggests that it may be a prosurvival factor for such cells in addition to RA FLS. B cells have a clear pathogenic role in RA as demonstrated by the efficacy of B cell depletion with anti-CD20 mAbs in the clinic [39]. It remains to be determined if IL-17 antagonists may directly reduce circulating and synovial B-cells in a similar way to current anti-TNF biologics or anti-B cell therapies [39], [40].

We describe a novel role for IL-17 in RA FLS survival via downregulation of FLS apoptosis. IL-17RA and IL-17RC mediated signaling and synoviolin expression may contribute to dysregulated RA FLS growth. Anti-apoptotic effects of IL-17-induced synoviolin are enhanced by TNF. Persistence of synovial Th17 synoviolin-expressing cells in close contact with B cells in germinal centre structures may further contribute to chronicity. These observations have important implications in the interaction between T cells and other stromal cells in the progression from initial synovial inflammation to hyperplastic pannus formation. Future IL-17 antagonists may limit both synovial inflammation and hyperplasia. Targeting IL-17 may improve efforts to control the chronicity of the disease, possibly in addition or following the control of inflammation with current cytokine inhibitors.

## Methods

### Ethics statement

The use of patient biological samples and this study was reviewed and approved by the local institutional review committee of the hospital of Lyon, and patients gave informed written consent.

### Isolation and culture of RA FLS

RA FLS were obtained from synovial tissue obtained from RA patients undergoing joint surgery who fulfilled the American College of Rheumatology criteria for RA [41]. Osteoarthritis (OA) FLS or dermal fibroblasts were obtained from the joint or the skin of patients with OA undergoing joint surgery; these cells were used as control cells for the RA FLS data. FLS or dermal fibroblasts were isolated by enzyme digestion and cultured in DMEM/10% FCS as described [3]. Cells beyond the third passage were >99% CD45 negative, and used between passages 4 and 9. Cultured RA FLS were treated with IL-17A or IL-17F 50 ng/ml (R&D Systems, Minneapolis, MN), IL-1β 0.1 ng/ml (Sigma-Aldrich), LPS 100 ng/ml (Sigma-Aldrich), TNFα 1 ng/ml (Biosource, Carmarillo, CA). IL-17RA or IL-17RC were antagonized with anti-IL-17RA mAb or anti-IL-17RC polyclonal goat Ab (R&D Systems). ERK, p38 and JNK MAPK were antagonized with PD98059 (50μM, chemicon)[42], SB203580 (5μM, chemicon)[43], or SP600125 (50μM, chemicon)[44].

### RNA interference

Gene-specific small interfering RNA (siRNA) corresponding to a 19-nucleotide sequence targeting 1623–1641 of human IL-17RA (IL-17RA siRNA, NM_014339), 985–1003 of human IL-17RC (IL-17RC siRNA, NM_153460) or human synoviolin (Synoviolin siRNA, NM_032431) were purchased from Dharmacon (Lafayette, CO, USA). A non-targeting siRNA (si*CONTROL*) was used as a control for non-sequence-specific effects. RA FLS (1×10^5^) were transfected with 0.5 µg IL-17RA, IL-17RC, synoviolin siRNA or si*CONTROL* siRNA duplexes using a human dermal fibroblast nucleofector kit, protocol U-23 (Amaxa GmbH, Cologne, Germany)[23].

### Apoptosis

RA FLS were treated with sodium nitroprusside (sigma) 0.01–0.5 µM for 24 h at 37°C. Apoptosis was analysed by annexin V expression (BD biosciences) by flow cytometry (Fluorescence activator cell sorter scan, Becton-Dickinson, Mountain View, CA) or fluorescence microscopy using an Axiovert 135 microscope (Zeiss) equipped with an AxioCam camera. Dead cells were excluded by PI staining. Apoptosis was also measured by a single stranded DNA (SS DNA) apoptosis ELISA kit (chemicon).

### Western Blot analysis

Synoviolin expression was examined by Western blot using antibodies against synoviolin (Abgent) [3] or β-actin (Santa Cruz, Cell Signaling Laboratories) as described previously [45]. Membranes were stripped and serially reprobed with antibodies against synoviolin and β-actin used as a loading control.

### Real-time RT-PCR Analysis

1 µg of RNA extracted using TRIzol reagent (Gibco BRL) was reverse transcribed using Thermoscript RT-PCR system (Invitrogen, California, USA) and PCR amplification performed on a LightCycler (Roche) using Fast-StartTM DNA Master SYBR Green I real-time PCR kit (Roche Molecular Biochemicals) [3]. Primer-specific nucleotide sequences for c-fos (Search-LC GmbH, Heidelberg, Germany), c-jun (Search-LC GmbH, Heidelberg, Germany)**,** p-65 (Search-LC GmbH, Heidelberg, Germany), β-actin (accession number: X00351, 5′-TGTCCCTGTATGCCTCTGGT-3′ and 5′-GATGTCACGCACGATTTCC-3′) or GAPDH (Search-LC GmbH, Heidelberg, Germany) were used and genes measured by quantitative real-time RT-PCR (RT-PCR).

### Chronic SCW-induced arthritis model

The mice and the arthritis model have been previously described in detail [30]. All in vivo mouse studies complied with national legislation and were approved by local authorities of the Care of Use of Animals of the University of Nijmegen, the Netherlands. Animals were housed under conventional conditions, water and standard laboratory chow were provided *ad libitum*. Specific pathogen–free C57BL/6 mice (males and females, ages 8–10 weeks; National Cancer Institute, Frederick, MD) were used as WT controls for the IL-17R^–/–^ mice. The IL-17R^–/–^ mice were obtained from Dr. J. J. Peschon (Amgen, Seattle, WA). Arthritis was induced in WT and IL-17R^–/–^ mice by the intraarticular injection of 25 µg of streptococcal cell wall (SCW) fragments in 6 µl of phosphate buffered saline into the knee joints. Chronic arthritis was induced by 5 repeated injections of 25 µg of SCW administered intraarticularly at 1-week intervals. On day 42, mice were killed by cervical dislocation and knee joints were isolated for histological analysis. The arthritis severity and inflammatory infiltrates were scored as previously described .

### Detection of synoviolin expressing cells

Mouse joints were fixed in 10% formalin, decalcified in formic acid and embedded in paraffin. Briefly for single staining, synoviolin expression was measured by immunohistochemistry using 12.5 µg/ml of a polyclonal rabbit antibody against synoviolin (Abgent). In negative control sections, irrelevant antibody (goat serum) was applied., and counterstained with haematoxylin [46], [47]. Cells positive for synoviolin were quantified by counting positive cells in ten consecutive high power fields (x 200). The number of positive cells per high power field was averaged and the results expressed as the number of synoviolin positive cells per mm^2^ using Histolab™ microvision version 5.9.2 [48]. For double staining, sections were incubated with a polyclonal rabbit antibody against synoviolin (Abgent) followed by biotinylated anti-rabbit immunoglobulins, and streptavidin-peroxidase (DAKO, Glostrup, Denmark). Peroxidase was developed by 3,3′ diaminobenzidine chromogen solution (DAB) (DAKO, Glostrup, Denmark). Goat polyclonal anti-mouse CD20 (4 µg/ml, Santa Cruz Biotechnology, Europe) and goat polyclonal anti-mouse IL-17 antibodies (10 µg/ml, R&D Systems Europe, London, UK) were followed by biotinylated anti-goat immunoglobulins (DAKO, Glostrup, Denmark) and streptavidin-alkaline phosphatase (DAKO, Glostrup, Denmark). Alkaline phosphatase was revealed using Vector Blue as chromogen (blue color; Vector Labs, Burlingame, CA, USA). In negative control sections, irrelevant antibody (rabbit or goat serum) was applied.

### In situ apoptosis and proliferation in chronic SCW arthritis

TUNEL staining was performed using an in situ death detection kit (Roche, Mannheim, Germany) according to the manufacturer's instructions. Synovial proliferation was determined by immunostaining using a specific Mab to the proliferation marker PCNA (Santa Cruz Biotechnology). Tunnel or PCNA-positive cells in murine synovial tissue samples were counted in a blinded manner in ten high-power fields per section, an average score was obtained and results expressed as the number of Tunnel or PCNA-positive cells mm^2^ using Histolab™ microvision version 5.9.2.

### Statistical analysis

Results are expressed as the mean ± SEM. Analysis was performed using the Mann Whitney U-test and *P* values <0.05 considered statistically significant. All data are the result of at least 3 separate experiments from 3 individual human RA donors.
